# Accuracy of Linear Measurements of Galileos Cone Beam Computed Tomography in Normal and Different Head Positions

**DOI:** 10.1155/2012/214954

**Published:** 2012-07-16

**Authors:** Mahnaz Sheikhi, Sajad Ghorbanizadeh, Mehrdad Abdinian, Hossein Goroohi, Hamid Badrian

**Affiliations:** ^1^Department of Oral and Maxillofacial Radiology, Dental School, Isfahan University of Medical Sciences, 8174673461 Isfahan, Iran; ^2^Dental Implant Research Center, Isfahan University of Medical Sciences, 8174673461 Isfahan, Iran

## Abstract

*Introduction*. The aim of this study was to determine the accuracy of linear measurements in dry human skulls in ideal position and different deviated positions of the skull. *Methods*. 6 dry human skulls were included in the study. Opaque markers were attached to alveolar bone. Buccolingual and mesiodistal distances and heights were measured in 5 different regions of either jaws using a digital caliper. Radiographic distances were measured in ideal, rotation, tilt, flexion, and extension positions of the skulls. The physical and radiographic measurements were compared to estimate linear measurement accuracy. *Results*. The mean difference between physical measurements and radiographic measurements was 0.05 ± 0.45. There was a significant difference between physical measurements and radiographic measurements in ideal, rotation, tilt, and extension positions (*P *  value < 0.05). *Conclusions*. The accuracy of measurements in GALILEOUS CBCT machine varies when the position of the skull deviates from ideal; however, the differences are not clinically significant.

## 1. Introduction

Cone Beam Computed Tomography (CBCT) has found its niche in different fields of dental practice during recent years [1–5]. Nowadays, the use of three-dimensional radiographies is increasing for diagnosis and treatment in dentistry. Single-slice CT and Multislice CT techniques were first introduced in this regard [6, 7]. The case against these two systems was that they exposed patients to high-radiation doses [8–10]. Thus CBCT was introduced to promise low-radiation doses together with adequate image quality, as well as fast image processing and lower costs [11, 12]. Consequently, the use of this technique dramatically increased in implant dentistry [2], maxillofacial surgery [1, 13], orthodontics [14], endodontics [15], and so forth. As different treatment approaches highly depend on the exact estimation of distance between anatomical landmarks and bone thickness, many clinicians tend to use the linear measurement capability of CBCT. Unfortunately, unwanted measurement errors may lead to catastrophic consequences like treatment failure [16, 17]. Since CBCT machines are not equipped with cephalostat, the skull might be in eccentric position during scanning procedure [18]. Previous studies have investigated accuracy of linear measurements in CBCT images using the NewTom 3G, Accuitomo, and other CBCT machines [17, 19–22], concluding that the linear measurement capability of these units is reliable for the structures closely associated with dentomaxillofacial imaging. Hassan et al. have evaluated the effect of patient's head position on linear measurement accuracy of NewTom 3G CBCT machine [23]. They declared that patient's head position does not influence linear measurement accuracy. Mischkowski et al. evaluated the geometric accuracy of scans obtained with Galileous CBCT device [24]. They reported that the cone beam device provides satisfactory information about linear distances. Also Ganguly et al. [25] evaluated the geometric accuracy of Galileous cone beam CT (CBCT). They declared the liner measurements between anatomical structures in the present of soft tissue are significantly accurate.

Although the newly introduced Galileous CBCT machine is reported to be one of CBCT dental devices with the lowest effective dose [26], its linear measurement accuracy has not been evaluated in different positions. Since it is probable that the patient's head deviate from true vertical or horizontal orientation during scanning procedure; which might adversely affect measurement accuracy [23], the aim of this study was to determine the accuracy of linear measurements in dry human skulls in ideal position and different deviated positions by simulating clinical relevant distances, using Galileous CBCT machine.

## 2. Materials and Methods

This is an experimental study on human skulls which was conducted in Isfahan University of Medical Sciences and approved by ethical committee of Isfahan Dental School Research Center.

### 2.1. Skull Preparation

6 dry human skulls were included in the study. The skulls were not identified by age, sex, or ethnicity. 5 regions were selected on each jaw; anterior, premolar, and molar regions of left and right sides of the jaw. In order to measure buccolingual and mesiodistal distances and height in each region, four points were determined using 1.5 mm rod-shaped gutta-percha (size# 40) opaque markers (Table 1): in the way that the first marker was glued to the embrasure of buccal alveolar crest of the studied region, the second marker to embrasure of lingual alveolar crest, perpendicular to the first marker, the third marker to the most apical region of buccal alveolar crest in the same direction with the first marker, and the fourth marker to the adjacent tooth's embrasure of buccal alveolar crest, next to the first marker.

### 2.2. Physical and Radiographic Measurement

The buccolingual, mesiodistal distances and heights of each region were measured two times by the first observer with one-week interval and once by a second observer, using a digital caliper (Guanglu, Taizhou, China). 

**Figure 1:**
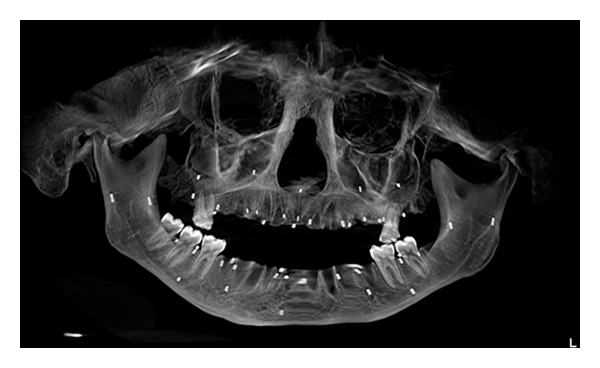
Panoramic view of the skull radiograph with 10 degree left tilt. Opaque gutta-percha markers are observed in the image.

**Figure 2:**
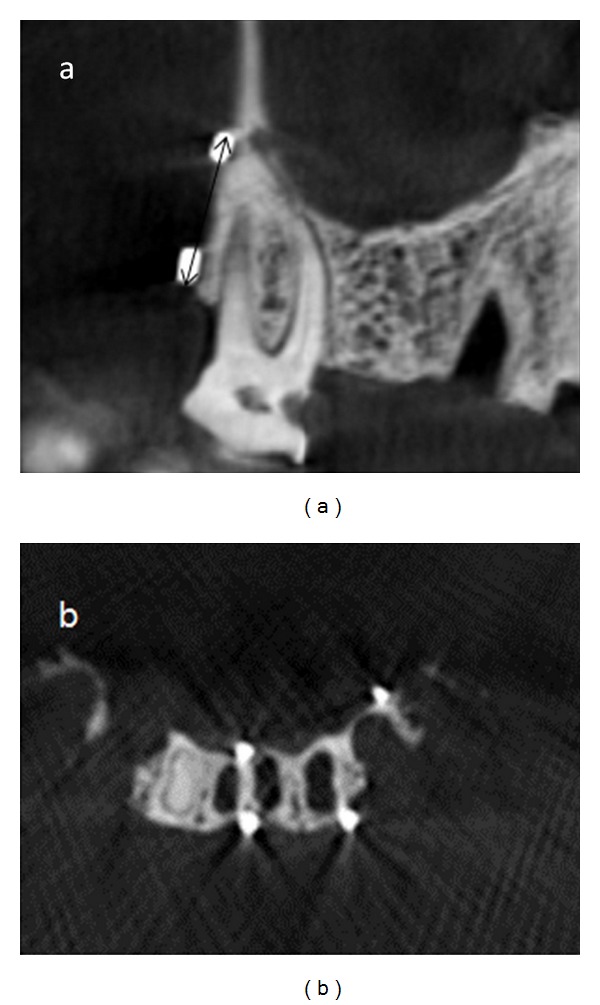
Examples of measured distances in different views. (a) Height: the arrow shows the measured distance; (b) buccolingual and mesiodistal length.

Then, the skulls were prepared for radiographic assessment. The images were taken using GALILEOS Comfort 3D imaging system (Sirona Dental Systems Inc., Bensheim, Germany). 5 different radiographs were provided for each skull in different positions: ideal position and positions with 10-to-15-degree rotation, 10-to-15-degree tilt, 10-to-15-degree forward tilt (flexion), and 10-to-15-degree backward tilt (extension). To reconstruct the temporomandibular joint space, a 1.5 mm-thick baseplate wax was placed between condylar process and temporal fossa. After guiding the jaw into the centric occlusion, the jaws were attached by an adhesive tape. A polyvinyl pipe was placed into the foramen magnum and attached to a camera tripod (Zeiss Universal Tripod FT6302, Oberkochen, Germany) with capability of lateral and forward-backward tilt and rotational adjustment, via a dial plate. To provide standard radiographs, the skull was held in the image field using the machine's occlusal bite block between teeth (in the way that the occlusal plane was perfectly horizontal; according to manufacturer's instructions). To ensure appropriate position of skulls, the system's light localizer, which displays the midsagittal line, was also used. For other positions of the skull, the camera tripod was adjusted to desired tilt or rotation. Then, imaging was performed at 7 mA (42 mAs) and 85 kVp, with 14-second scan time and 270° rotation. Each scan produced 200 projections in a 15 × 15 × 15 cm field of view. A charge-coupled device detector, with 1024 × 1024 matrix and 0.15 mm voxel size, was used to detect the images. Images were saved in SVG file format and reconstructed using GALAXIS Viewer software ver. GAX5 (Figures 1 and 2). Afterwards, the radiographic distances were measured twice by the first observer with two-week interval and once by the second observer. It has to be added that the measured distance was from the end of one marker to the end of another. The physical and radiographic distances, measured in each region, are illustrated in Table 1.

### 2.3. Statistical Analysis

The statistical analyses were performed by SPSS software version 18. Intraclass Correlation Coefficient (ICC) was used to analyze intraobserver and interobserver reliability of measurements (*α* = 0.05). Wilcoxon test was used to compare physical and radiographic values of different measurements (*α* = 0.05); the less the differences between physical and radiographic measurements are, the more accuracy of radiographic measurements will be. One-sample *T*-test was used to compare the differences with the acceptable 0.5 mm mean absolute error (*α* = 0.05). Univariate analysis of variance was used to assess the difference between radiographic and physical measurements, considering the confounding variables. Post hoc Tukey test was performed to determine the significant differences.

## 3. Results

Due to severe bone resorption, the distances in the mandibular left premolar and molar regions in one skull and also mandibular right premolar and molar regions in another skull were not separately measured, reducing the whole measurements to 174. According to ICC values, Interobserver correlations for radiographic measurement and for physical measurements were both 0.996 (*P* value < 0.001). Intraobserver correlation was 0.995 for radiographic measurements (*P* value < 0.001) and 0.996 for physical measurements (*P* value < 0.001). The mean difference between physical measurements and radiographic measurements in the present study was 0.05 ± 0.45.

Table 2 illustrates the overall accuracy of measurements. There was a significant difference between physical measurements and radiographic measurements in ideal, rotation, tilt, and extension positions.

Table 3 shows the accuracy of measurements in different measured aspects. Accuracy of measurements for height was significantly lower than physical measurements in all positions (*P* value < 0.05).

Table 4 shows the difference between radiographic and physical measurements considering the confounding variables. Tukey HSD showed that the accuracy of measurements on skull number 5 was significantly lower than skulls number 2, 3, 4 and 6 (*P* value = 0.022, 0.001, 0.006, and 0.001, resp.). Also, accuracy of measurements in rotation and flexion positions was significantly lower than ideal position (*P* value = 0.042, 0.043 resp.). There was no significant difference among accuracy of measurements in molar, premolar, and central regions (*P* value < 0.05). Accuracy of measurements in the left sides of skulls was significantly lower than the right side (*P* value = 0.01). There was no significant difference between upper and lower jaws in terms of accuracy of measurements (*P* value = 0.115).  Table 5 compares the absolute mean difference between each radiographic position and 0.5 mm acceptable error. This table shows that the accuracy of measurements in all cases was above 0.5 mm.

## 4. Discussion

The patient's position might deviate from ideal before imaging procedure [18]. It is important to determine whether the accuracy of measurements decreases or remains unchanged, when the patient's head position changes. The aim of this study was to determine the accuracy of linear measurements in dry human skulls in different positions of the skull by simulating clinical relevant distances. 

In the present study, soft tissue was not simulated to prevent its probable confounding effect on accuracy of measurements [27]. Main errors in patient's head position (tilt, extension, flexion, and rotation) were evaluated. Lateral displacement was not assessed [21], since this position error less likely occurs. Due to the probable effect of teeth situation and the type of jaw, both mandible and maxilla were investigated in five areas. Since radiographic units should be able to measure height, mesiodistal length, and buccolingual length of implant treatments [28], all of these distances were included in the present study. The rod-shaped opaque markers would let the observers more easily and accurately measure the distance between the end of one marker and the end of another. To make sure that the measurements were accurate, two observers measured the distances. The intraobserver and inter-observer correlations above 0.95 show that the accuracy was acceptable. The results of the study showed that there were some differences in measurement between normal and deviated positions in some cases; however, since the average difference was less than 0.5 mm, they are not considered clinically significant [27].

Comparison of the accuracy of radiographic measurements between ideal and deviated positions showed that significant difference exists in rotation and flexion positions, which may suggest that these positions are the most effective deviations for measurement accuracy. Hassan et al. [23], in assessment of accuracy of measurements on dry human skulls, found no significant difference between different positions of skulls. This contrast with the present study is probably due to lower number of measurements and longer distances in that study. Ludlow et al. [29] declared that no significant difference was observed between different positions. This contrast might be due to different distances that were measured in these studies, as well as different study designs.

The high rates of standard deviation of differences between physical and radiographic measurements in the present study suggest that more than a few factors are affecting the accuracy of measurements. 

There are a number of reasons that may justify differences in the accuracy of measurements. Predominant artifacts in CBCT imaging including noise, scatter, extinction artifact, beam hardening, exponential edge gradient effect, aliasing artifacts, and ring artifacts [30] may cause difficulties in detecting the exact situation of objects in a CBCT output image which leads to inaccurate measurements. 

Distortion also can cause errors in length measurements. The rotation of CBCT unit with wobble pattern is a probable source of distortion. Moreover, the anatomical distortions, a function of shape and orientation of the structures, can cause distortions as well. [29] Results of the present study showed that accuracy of measurements is affected by skull type. This suggests that CBCT units can be enhanced by features like a posterior position adjustment [31], letting the practitioner consider anatomical asymmetries and differences; however, distortion caused by anatomical asymmetry is not always distinguishable from radiographic distortion [29]. 

On the other hand, although some deviated skulls showed severe distortion in the panorama view of GALAXIS software in the present study, the distances between markers could still be accurately measured in cross-sectional, tangential, and axial views. This is partly due to the fact that CBCT software lets the practitioner choose not only the orientation of reconstructed image layer [29] but also the image plane that cross-sectional, tangential, and axial views are expected to display; however, failure in appropriate setting will lead to difficulties in measuring distances. For instance, to see acceptable cross-sectional image planes, one should previously set the image plane in panorama view. If the practitioner fails to do so, cross-sectional display will not be appropriate for measurements. As an observation of the present study, sometimes the markers that were precisely placed in buccal and lingual embrasures of a tooth could not be displayed at the same time in cross-sectional view of the software, even with changing the image plane in panorama or the orientation of reconstructed image layer, which implies a shortcoming in the unit or the software and should be corrected. 

The mean difference between physical measurements and radiographic measurements in the present study was 0.05 ± 0.45 mm. The measurement points were selected using mouse cursor. Ludlow et al. [29] who used the same method for selection of the points, reported 0.29 ± 0.20 mm mean difference. Marmulla et al. [17] however, used computer algorithm to localize measurement points with pixel fractionalization. They reported 0.13 ± 0.09 mm mean difference. These differences are due in part to the different study designs, measurement techniques, and CBCT units used. Moreover, Leung et al. [22] who measured alveolar bone height reported that the accuracy was 0.6 mm. These contrary results can be explained by the fact that determining anatomical landmarks is more difficult than opaque markers.

 The mean absolute error of Galileos CBCT machine was estimated to be 0.26 ± 0.18 mm by Mischkowski et al. [24]. The difference between this study and the present study is probably due to different investigated areas and distances measured.

Lund et al. [21] reported that the measurements in CBCT were very accurate and the absolute mean difference was even less than voxel size. This could be explained by the nature of specimens. In fact, Lund et al. used an object consisting of Plexiglass plates, while the present study was performed on human dry skulls.

In the present study for evaluating the effect of different head position all skulls were tilted to the left side. It may be the reason of significant difference of between the left and right side.

The manufacturer of Galileos CBCT machine claims that the accuracy of length measurements is ±0.15 mm; however the results of the present study showed that this accuracy depends on numerous criteria and is not always in the above range. We suggest that the same study with larger number of skulls be conducted so that more criteria such as aspect of measurement can be included in univariate ANOVA. Moreover, it is recommended to simulate soft tissue attenuation in one separate group to assess its probable effect. 

In conclusion, the present study demonstrates that the accuracy of measurements in Galileos CBCT machine varies when the position of skull deviates from ideal; however the reduction in accuracy could be clinically considered insignificant.

## Figures and Tables

**Table 1:** Different physical and radiographic measured distances in molar, premolar, and anterior regions.

	Physical	Cross-sectional	Axial	Tangential
Buccolingual length	Between 1st and 2nd markers	+	+	
Height	Between 1st and 3rd markers	+		+
Mesiodistal length	Between 1st and 4th markers		+	+

1st marker: placed on the embrasure of buccal alveolar crest.

2nd marker: placed on embrasure of lingual alveolar crest, perpendicular to the first marker.

3rd marker: placed on to the most apical region of buccal alveolar crest in the same direction with the first marker.

4th marker: placed on the adjacent tooth's embrasure of buccal alveolar crest, next to the first marker.

**Table 2:** Mean difference between physical measurements and radiographic measurements in different positions.

Position	Mean difference ± SD (mm)	*P* value
Ideal	0.04 ± 0.44	0.032
Rotation	0.14 ± 0.52	<0.001
Tilt	0.10 ± 0.53	0.001
Flexion	0.10 ± 0.61	0.107
Extension	0.04 ± 0.44	0.053

**Table 3:** Mean difference between physical measurements and radiographic measurements in different positions, considering the measured aspect.

Position	Aspect	Mean difference ± SD (mm)	*P* value
	Buccolingual	0.01 ± 0.13	0.431
Ideal	Height	0.15 ± 0.58	0.022
	Mesiodistal	0.02 ± 0.49	0.350

	Buccolingual	0.17 ± 0.46	0.007
Rotation	Height	0.23 ± 0.68	0.024
	Mesiodistal	0.03 ± 0.38	0.041

	Buccolingual	0.18 ± 0.49	0.458
Tilt	Height	0.12 ± 0.63	0.002
	Mesiodistal	0.01 ± 0.44	0.043

	Buccolingual	0.13 ± 0.48	0.979
Flexion	Height	0.18 ± 0.89	0.037
	Mesiodistal	0.09 ± 0.32	0.317

	Buccolingual	0.13 ± 0.50	0.559
Extension	Height	0.16 ± 0.38	0.004
	Mesiodistal	0.01 ± 0.43	0.883

**Table 4:** Assessment of difference between radiographic and physical measurements: univariate ANOVA and resulting *P* values.

Source	Factor	*P* value
Position		0.036
Skull		<0.001
Region	Molar, premolar, central	0.118
Jaw	Maxilla, mandible	0.115
Side	Left, right	0.010
Skull ∗ region		0.070
Skull ∗ side		0.006
Region ∗ position		0.098

**Table 5:** Comparison of mean differences with 0.5-mm absolute error: One-sample *t*-test.

Position	Absolute value of difference ± SD (mm)	*P* value
Normal	0.05 ± 0.45	<0.001
Rotation	0.14 ± 0.52	<0.001
Tilt	0.35 ± 0.41	<0.001
Flexion	0.32 ± 0.32	0.002
Extension	0.37 ± 0.50	<0.001
